# A Method for Assessing Working Memory in Rats Using Controlled Virtual Environment

**DOI:** 10.17691/stm2024.16.3.02

**Published:** 2024-06-28

**Authors:** A.V. Lebedeva, S.A. Gerasimova, M.I. Yashanova, A.V. Naumov, A.A. Ivanov, D.A. Karchkov, O.V. Martynova, A.E. Malkov, T.A. Levanova, A.N. Pisarchik

**Affiliations:** PhD, Associate Professor, Department of Neurotechnologies, Institute of Biology and Biomedicine; National Research Lobachevsky State University of Nizhny Novgorod, 23 Prospekt Gagarina, Nizhny Novgorod, 603022, Russia; PhD, Researcher, Research Laboratory for Perspective Methods of Multidimensional Analysis, Institute of Information Technologies, Mathematics and Mechanics; National Research Lobachevsky State University of Nizhny Novgorod, 23 Prospekt Gagarina, Nizhny Novgorod, 603022, Russia; Assistant, Department of Biology; Privolzhsky Research Medical University, 10/1 Minin and Pozharsky Square, Nizhny Novgorod, 603005, Russia; Research Assistant, Research Institute of Neurosciences; National Research Lobachevsky State University of Nizhny Novgorod, 23 Prospekt Gagarina, Nizhny Novgorod, 603022, Russia; Research Assistant, Research Institute of Neurosciences; National Research Lobachevsky State University of Nizhny Novgorod, 23 Prospekt Gagarina, Nizhny Novgorod, 603022, Russia; Senior Teacher, Department of Mathematical Support and Supercomputer Technologies, Institute of Information Technologies, Mathematics and Mechanics; National Research Lobachevsky State University of Nizhny Novgorod, 23 Prospekt Gagarina, Nizhny Novgorod, 603022, Russia; PhD, Senior Teacher, Department of Electrodynamics, Faculty of Radiophysics; National Research Lobachevsky State University of Nizhny Novgorod, 23 Prospekt Gagarina, Nizhny Novgorod, 603022, Russia; PhD, Senior Researcher, Research Institute of Neurosciences; National Research Lobachevsky State University of Nizhny Novgorod, 23 Prospekt Gagarina, Nizhny Novgorod, 603022, Russia; PhD, Associate Professor, Department of System Dynamics and Control Theory, Institute of Information Technologies, Mathematics and Mechanics; National Research Lobachevsky State University of Nizhny Novgorod, 23 Prospekt Gagarina, Nizhny Novgorod, 603022, Russia; PhD, Chair in Computational Systems Biology, Center for Biomedical Technology; Universidad Politécnica de Madrid, Madrid, 28223, Spain

**Keywords:** virtual environment, T-maze, working memory in rats

## Abstract

**The aim of the study** is to develop an experimental method to effectively assess the working memory in rats. The method uses a state-of-the-art controlled virtual environment with a virtual maze. The setup includes a treadmill for rodents, a fixation system, a dome for displaying virtual environment, and a control unit.

**Materials and Methods:**

**Biological part of the investigation**

In our study, young healthy Wistar rats aged 6–7 months were used. The initial stage involved habituating the experimental animals to the experimenter over a period of two weeks. The habituation process was conducted in several successive steps. First, the rats were acclimated to wearing a jacket, which is part of the apparatus that holds the animal in the experimental setup. Next, they were familiarized with the fixation system. Following this, the rats were introduced to the treadmill (a sphere), and finally, they were acclimated to the entire setup. Subsequently, the rats were gradually habituated to the virtual maze and the associated reward system through positive reinforcement. This approach helped minimize stress and facilitated their adaptation to the new conditions. The second stage involved exploring the virtual space and learning the features of the virtual maze, including walls, turns, and the end goal. During the learning phase, the animals received positive reinforcement in the form of sugared water from the automatic water dispenser for correctly performed tasks. To navigate the T-maze, the rats used visual cues such as wall color and figures on the wall. At this stage, the rats learned to use virtual space to achieve their goals. Once the rats showed evident progress in learning the virtual environment, we implemented a protocol to assess their working memory. This assessment was based on the time it took for the rats to find the maze arm that provided positive reinforcement.

**Engineering part of the investigation**

The animal is positioned on a foam plastic sphere with a 30 cm radius, using a custom device that allows its head and paws to remain mobile. Bearing fix the sphere in place, enabling the rat to rotate freely around its vertical axis. The rat’s forward and backward movements cause the sphere to rotate, simulating a treadmill. The sphere’s movements are detected by two infrared sensors (adapted from optical LED mice with USB interfaces) and transmitted to a computer, which generates an image of the virtual environment — a maze with landmarks on its walls. The virtual environment, created using the Unity Real-Time 3D Development Platform, is projected onto a custom-designed dome display containing the sphere and the lab rat. The setup provided the rat with a 360° field of view.

**Conclusion:**

In our study, we present a setup that includes a projector, a dome display, a sphere (treadmill), a virtual T-maze, motion capture sensors, systems for securing animals to the sphere, and positive reinforcement delivery systems. We have developed an optimal protocol for immersing laboratory animals into a virtual environment and evaluating their cognitive functions, particularly working memory. The application of virtual reality in biological experiments enables more precise control over study conditions and allows for the creation of highly accurate and realistic behavioral protocols to assess cognitive functions in animals. This approach enhances our understanding of the mechanisms underlying working memory and their relationship with behavioral processes in rats and other animals.

## Introduction

Rapid advances in engineering and computer technology are setting new standards for behavioral experiments with animals. Traditional methods, such as physically constructed T-mazes, are not always optimal for exploring memory and behavioral aspects. One potential issue is the risk of mechanical damage resulting from the animal’s vigorous head movements, especially if the head has implanted arrays. Such damage may also arise from the animal’s head contacting maze walls or other objects, which can confound experimental results and lead to unreliable conclusions [1, 2].

In light of these factors, researchers are developing more advanced systems to minimize mechanical injuries and eliminate artifacts. One promising approach is the use of virtual environments for conducting experiments [1–4]. These systems ensure reliable animal fixation to prevent discomfort while allowing the animal to move freely forward and even turn around. Full immersion into the virtual environment necessitates a panoramic view, enabling the animal to interact fully with its surroundings and turn its head as needed. This approach allows researchers to create controlled experimental conditions and study memory functions and other aspects of animal behavior with greater precision. Additionally, the virtual environment enables precise control over input data and the setting of complex and realistic tasks, leading to more accurate and reliable experimental results.

### Systems of virtual reality for investigations on rodents

The system, most commonly used for investigations on rodents usually consists of a display screen (on which virtual reality is projected) and a treadmill, on which the animal runs. The position of the animal body is fixed by fastening devices. There are several ways of animal fixation: body fixation [1, 5] or head fixation [6, 7]. The body can be fastened in different ways: rigid fixation, when the animal can move only in one direction, or fixation with a pivot lever allowing a free 360° rotation around its axis [4]. Head fixation gives the possibility to use methods for analyzing neuronal activity that require high stability. However, retaining animals in virtual reality systems limits the sensory information they receive. For example, head fixation hinders natural movements and completely excludes vestibular signals, which are important for spatial orientation of the animal. Thus, there is a mismatch between vestibular, proprioceptive, and visual signals.

Aronov et al. [4] have shown that only unrestricted rotation of the animal body in the visual virtual reality conditions ensures the formation of 2D patterns of neuronal activity in the entorhinal-hippocampal system similar to those observed in real life. A treadmill usually consists of a polystyrene sphere from or a similar material [6]. The mass of the sphere must be 1.5 times greater than that of the experimental animal. This ensures that the animal applies the same force to accelerate the sphere movement as it would to accelerate its body on the ground [5]. The animal is secured so that its movements rotate the spherical treadmill. The treadmill rotation is measured by motion sensors located near the treadmill (often 1 or 2 optical computer mice). A signal is fed to the computer, which generates and updates the virtual reality. These spherical treadmills allow for the use of two-dimensional virtual realities. However, some investigators apply rolling belts or cylindrical treadmills, enabling them to implement only one-dimensional virtual realities.

Several monitors [7] or a projector with a screen [3] are employed to visualize the virtual reality. The displays vary by the field of view. The most effective monitors have a 270–360° field of view, allowing full immersion of the animal into the virtual reality. This condition is considered especially important for rodent immersion since the field of view in rats covers 300° horizontally and from 45 to 100° vertically [3, 8]. Panoramic displays are usually toroidal or cylindrical screens, on which a virtual picture is presented using a projector and system of mirrors.

There is another variant of the system when the animal is placed inside a box, and virtual reality is projected onto the walls and floor [9]. In this setup, the animal can move freely within the box. However, these systems lack a closed loop between sensory stimulation and actions of the animal. Despite the absence of vestibular motion stimuli, activation of place cells and theta-rhythmicity in the hippocampus have been detected in these systems [9]. Visual information alone was sufficient for localized excitation in 25% of the place cells and maintaining the thetarhythm (although with significantly decreased power). Additional information related to motion was required to excite the remaining 75% of the place cells in the hippocampus.

One more important aspect is the application of color stimuli. Rodents show the highest sensitivity to light with 370 and 510 nm wavelength. Therefore, it is beneficial to include a green spectrum (500 nm) in the virtual reality environment [6]. Various virtual mazes are used to study memory function disorders. Radial 8-arm mazes, T-mazes, Y-mazes are widely used to evaluate of learning and working or long-term memory as classical neurobehavioral tests [10]. These methods are based on the rodent instinct to explore new places in combination with food reinforcement. However, advancements in current technologies have made it possible to transfer mazes to virtual reality and improve the methodology for studying memory and spatial navigation.

A colored wall T-maze with a specified direction of the turn to the desired arm is used more often. Besides, a lag period is introduced between the presented stimulus and the animal turn to the correct compartment of the maze [11, 12]. Currently, most virtual systems for rodents operate with visual stimulation only, but future plans include creating multisensory systems. These will allow researchers to improve investigations and obtain more accurate neuronal patterns in the animal brain during cognitive tasks [3, 13, 14].

### Types of memory investigated with the help of virtual systems

Three main types of memory are distinguished [15, 16]: sensory, short-term and longterm, each with its own subcategories. Sensory memory processes signals coming from the environment, acting as the border between perception and memory [17]. Short-term (working) memory provides storage for a small volume of information over a short period of time. Working memory is a system for temporary holding information and manipulating it to perform a large number of complex tasks. It is responsible for selecting and employing repetition strategies, serves as a global workspace, and directs information for long-term storage and retrieval. Short-term memory is further subdivided into auditory, visual, tactile, olfactory, taste, interoceptive and spatial memory [18].

Long-term memory is divided into explicit (declarative) and implicit (non-declarative) memory. Explicit memory includes semantic (accumulated knowledge about the world) and episodic memory (recollection of personally experienced events). Explicit memory involved voluntary and conscious reproduction of information, whereas implicit memory involves the retrieval of information through actions [16, 19].

One of the main parts of the brain responsible for memory mechanisms is the hippocampus. It plays a key role in the primary formation and memory storage. Recollections initially formed in the hippocampus gradually stabilize in the cortex of the cerebral hemispheres for a long-term storage. Important information from the hippocampus first goes to the anterior medial thalamus and then to the hemispheres [16, 20]. In the present study, we evaluated the mechanisms of remembering a virtual T-maze by experimental animals (rats) during their fixation by a fastening system and an original jacket.

**The aim of the study** is to develop an experimental method for evaluating working memory in rats using an advanced experimental setup. This setup includes a controlled virtual environment (a virtual maze), a treadmill for the rodents, a fixation system, a dome for displaying the virtual environment, and a control unit.

## Materials and Methods

### Biological part of the investigation

The study was carried out in compliance with the ethical principles established by the Bioethical Committee of the Institute of Biology and Medicine of Lobachevsky National Research University of Nizhny Novgorod. Our work was guided by Order No.199n “On the Approval of the Rules of Good Laboratory Practice” (Russia, 2016), the International Guiding Principles for Biomedical Research Involving Animals (CIOMS and ICLAS, 2012), and ethical principles of the European Convention for the Protection of Vertebrate Animals used for Experimental and Other Scientific Purposes (Strasbourg, 2006).

We used young healthy, outbred Wistar rats (males, 350–450 g, n=11) aged 6–7 months, in our study. In the first stage, the animals were habituated to the experimenter (the coauthor of this publication) for 2 weeks. Then, each separate element of the system was gradually introduced to the animals to help them get accustomed to it. All these steps of habituation are described in detail in the “Results” section. The sequence of all habituation steps is presented in the table in the biological part of the investigation.

**Table:** Stages of rodent habituation to the setup elements and T-maze

Stage 1	Stage 2	Stage 3	Stage 4	Stage 5
2 weeks	10 days	minimum 7 days	minimum 7 days	minimum 7 days
Habituation to experimenter (handling)	Habituation to the fixing jacket	Habituation to the system of fixtures	Habituation to the full kit and sphere	Habituation to the entire setup and virtual T-maze

### Engineering part of the investigation

**Figure 1.**
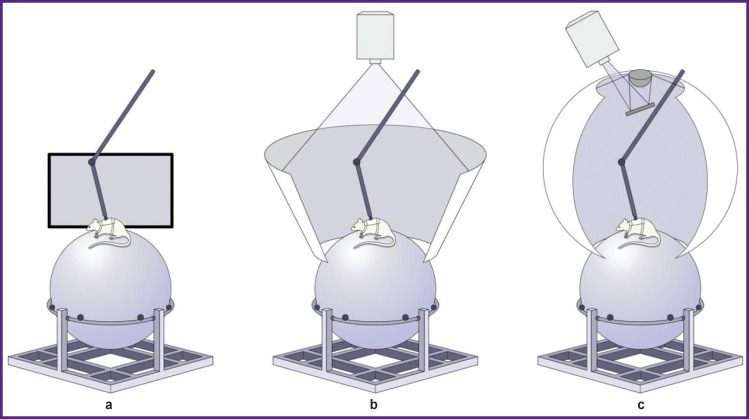
Variations of the setup for creation of virtual environment: (a) the maze is demonstrated to the rats using a display screen; (b) the maze is projected on a cone display; (c) the maze is projected on the spherical display

The laboratory setup is presented in Figure 1. The system is composed of the following main parts: a sphere (treadmill), upper fixation, lower fixation, hoop, fixations directly for the animal, projector (or mirror), and display. We considered three options in our work.

**Figure 2.**
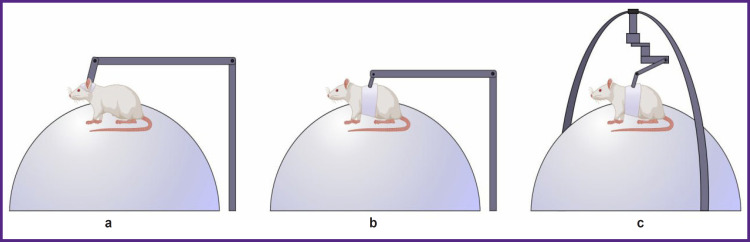
Variations of animal fixtures: (a) rigid head fixation; (b) rigid body fixation; (c) flexible body fixation

The first option, in which the maze is demonstrated to the animal on the screen, is presented in Figure 1 (a). In this case, rigid fixation of the animal head and body is necessary (Figure 2 (a), (b)), due to the small angle of view. One significant drawback of this setup is an open space at the sphere height, which causes the tested rats to experience stress, experienced by “chirping” and refusal of reward treats. Investigations in this setup require a longer habituation protocol and specific conditions, such as complete darkness in the laboratory, to minimize stress.

In Figure 1 (b), the maze is projected onto an especially manufactured cone display measuring 70×70×140 cm. This setup significantly reduces the level of stress in the experimental animals as a rats in an enclosed space, which is more natural for them since they are tunneling animals. This option is also relatively simple and does not require special materials for projection.

In Figure 1 (c), the image is projected onto a sphere-shaped display through a special lens, the construction of which is described in [3]. This option is the most labor-intensive, but it places the animal in a fully enclosed space.

The treadmill is a sphere from foam plastics with a 30 cm radius. The maze was designed using Unity Real- Time 3D Development Platform. The animal is secured on the sphere with an original device, allowing its head and paws to move freely. Flexible and rigid fixation can be applied to the animal head or body (Figure 2).

Rigid head fixation is necessary to control the rat movements when the field is insufficient field (Figure 2 (a)). In our work, we carried out such investigations to develop the habituation of the animal to the sphere and maze. The fixation shown in Figure 2 (b) is considered preferable since it is more natural and minimizes the stress. However, with rigid body fixation, the animal cannot turn, causing it to move only forward and restricting its turns. This type of fixation is also required to control the animal turns due to a field of view less than 360°. In our work, we used flexible fixation (Figure 2 (c)) with the help of bearings, which allowed the animal to rotate freely around the vertical axis.

The animal back-and-forth movements cause the sphere to rotate, similar to a treadmill. Movements of the sphere were captured by two infrared sensors (from standard optical USB mice) and sent to the computer, which generated the image of a virtual environment (the maze with landmarks). The virtual environment was projected onto an especially designed dome display, inside which there is a sphere with the tested rat. At this stage, the rat field of view was 360°.

## Results

In the process of our investigation, an experimental setup of virtual reality for rats has been created. Each element of the system was tested and examined in interaction with the experimental rats. We designed original fabric jackets to provide maximum comfort and reliable fixation. A system of fixtures and a sugared water presentation method have been proposed, and an effective protocol (the sequence of actions) for habituating the animals to the virtual environment has been also developed. The protocol of investigating working memory in rats was developed based on this experimental setup.

### Jackets for rat fixation

**Figure 3.**
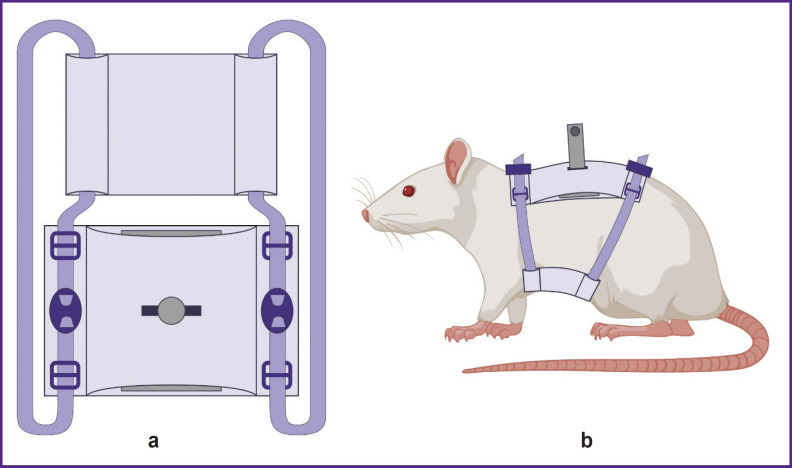
Schematic representation of the jacket for holding the experimental animal in the virtual reality system: (a) a general view of the jacket inside and outside; the central part consists of the fabric rectangles; fabric ribbons serve as lateral fixtures for securing the animal; (b) a view of the jacket on the experimental animal

The first element of the system was original fabric fixators, or jackets, for holding the rats in the setup (Figure 3). Rats acclimated to the jackets over 10 days until comfort was achieved.

### Fixations for rat holding and final view of the experimental setup

**Figure 4.**
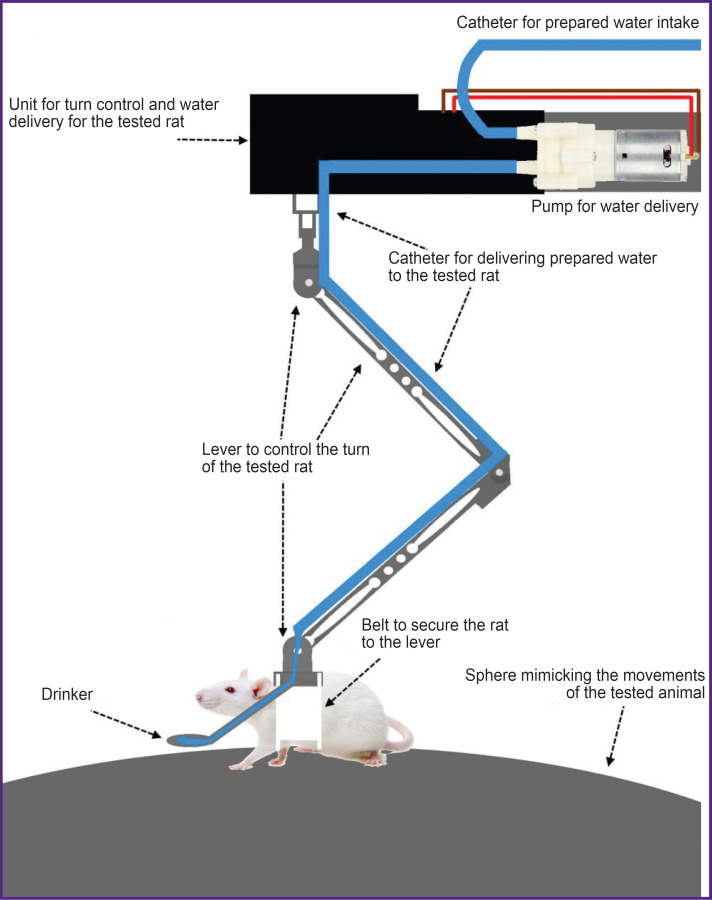
Schematic view of the developed fixture for the tested rat on the sphere and positive reinforcement presentation of sugared water from the drinker

**Figure 5.**
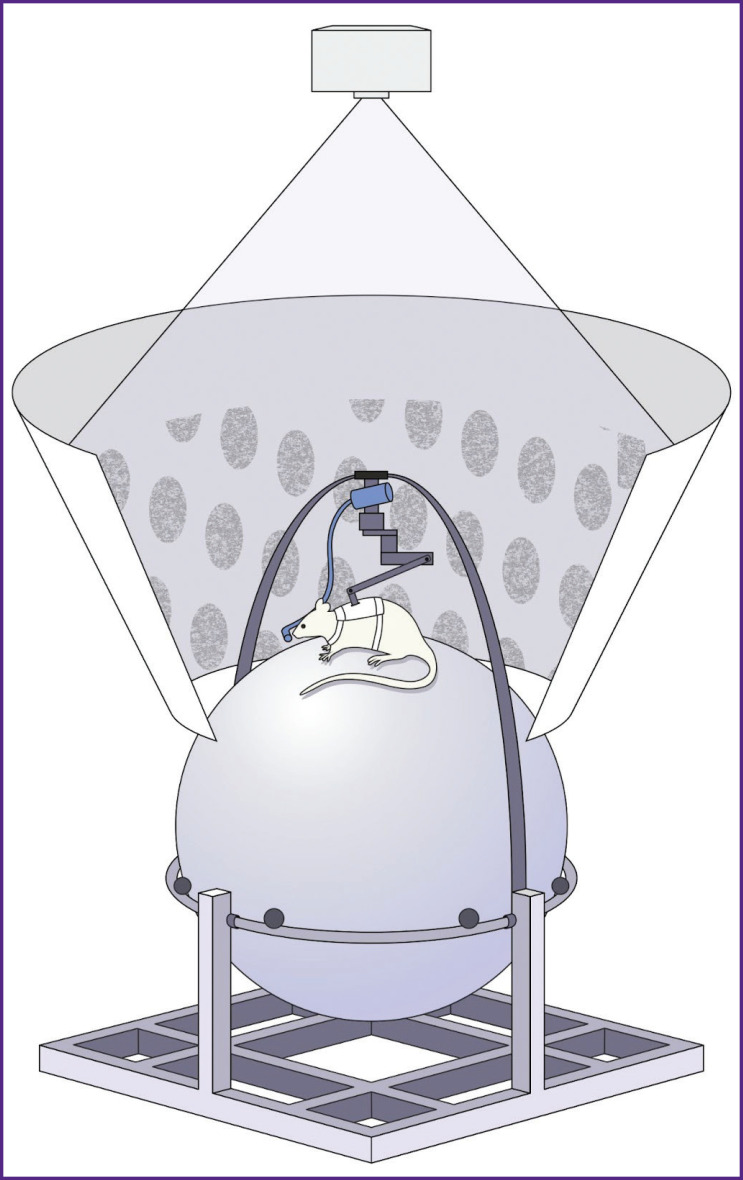
General view of the setup with the tested rat in the virtual maze The treadmill (sphere) is attached to the aluminum profiles. The rat motion sensors are mounted on the hoop on the bottom of the sphere. The tested rat in the fixing jacket is placed on the upper central part of the sphere with a fixture, which is connected with the jacket and half of the hoop on top directly with the entire setup for free movement. The virtual T-maze is projected to the cone display using a projector with a pattern of grey circles on the walls. If the rat turns correctly, it receives sugared water from the tube via the water supply system attached to half of the hoop from above

Once the rates were comfortable in the jackets, we began habituating the animals to the sphere (treadmill), then to the system of fixtures (Figure 4), and finally to the complete setup (Figure 5).

### Development of virtual T-maze

**Figure 6.**
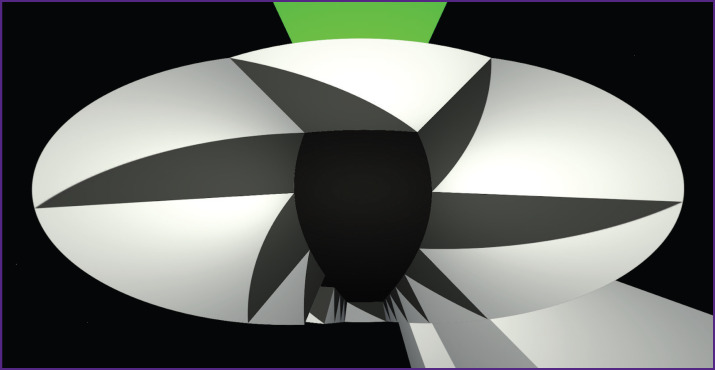
Virtual reality image for the tested rats inside the 360° cone display with a green tower as an endpoint landmark

A virtual environment (the T-maze) was developed using the Unity Real-Time 3D Development Platform, a tool for creating computer games. This virtual T-maze was projected onto a specially designed dome display, inside of which was the sphere with the tested rat. The field of view in this setup was 360° (Figure 6).

**Figure 7.**
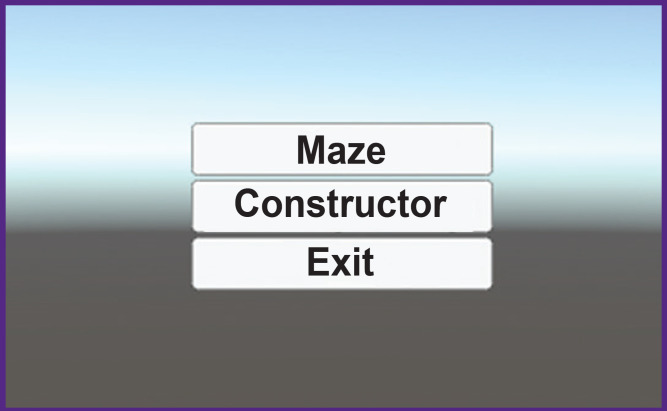
User interface of the first page of the virtual maze program

Two optical sensors from computer mice were used to capture movements of the experimental rats on the sphere. The middleware program sensed the delta movements from each mouse and transferred it to the virtual maze. The middleware is a console application necessary for splitting the input data from optical sensors and sending them to the virtual maze. The maze used these delta values to determine the distance the animal should be moved within the maze. Two sensors were required to accurately read the sphere turns in any directions. The virtual maze had a user interface, with the initial display shown in Figure 7.

**Figure 8.**
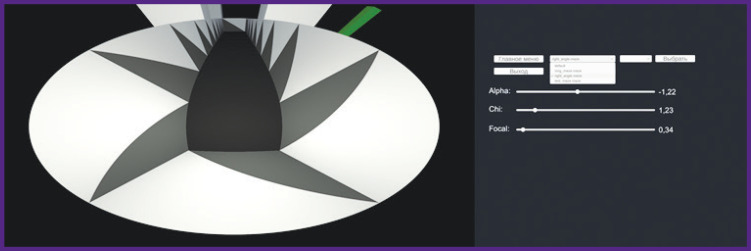
Virtual maze image (*left*) and display of the second monitor screen necessary for program control (*right*)

The button “Maze” can be selected to enter the virtual maze. The button “Constructor” serves to construct different maze variants. “Exit” denotes exit from the interface. When the button “Maze” is clicked, the transition of the scene occurs and the base maze image is displayed (Figure 8). The program is designed for two monitors: one as a projector the other for program control.

**Figure 9.**
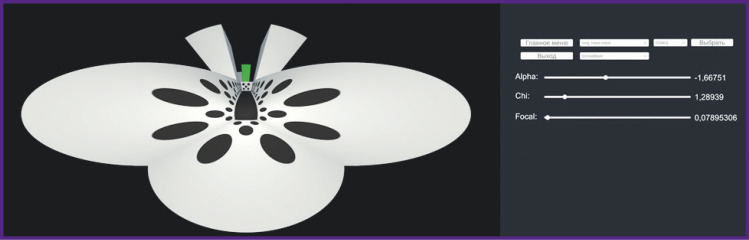
Illustration of the changed shape of the virtual maze (*left*) and display of the second monitor screen necessary for program control (*right*)

It is possible to change the parameters of camera displaying, which is necessary to adjust the output of the projector (Figure 9).

**Figure 10.**
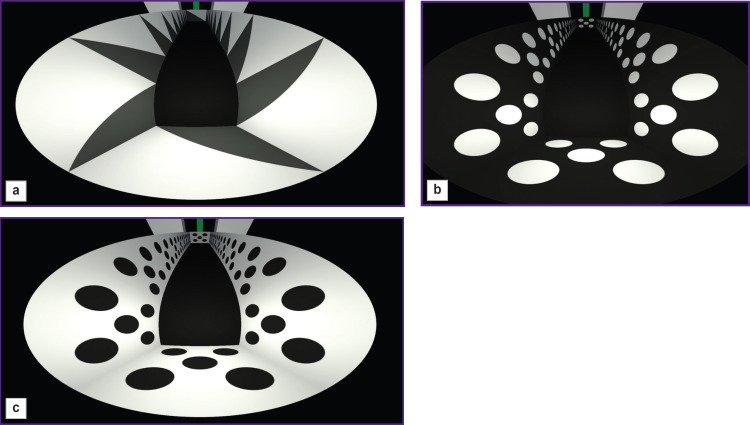
Variants of different element shapes on the virtual maze walls: (a) white walls with grey triangles; (b) black walls with white circles; (c) white walls with black circles

In Figure 10, various shapes and colors of the virtual maze walls are presented, allowing for the development of different experimental protocols to explore the memory processes in laboratory animals.

### Development of the protocol for evaluation of the working memory in rats using the virtual maze

In the next stage, gradual habituation to the virtual maze included implementing a system of reward. To stimulate interest in water as a reward (sugared water in our case), animals were deprived of plain drinking water 2 weeks before the experiment. The exploration of the virtual space and learning the landmarks of the maze (such as walls, turns, the endpoint) constituted the second stage. The protocol from the work [12] served as the foundation and was adapted to suit our investigation.

**Figure 11.**
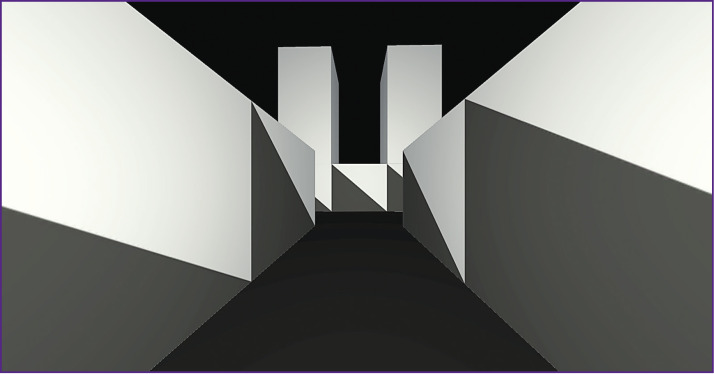
Illustration of the developed virtual T-maze with a central track, left and right turns and two towers at the end of the 2D projection

Fully equipped animals were placed on a treadmill with a linear track within the virtual T-maze (Figure 11). The T-maze was chosen for its ability to assess animal working memory based on their ability to remember the desired arm of the maze in search of positive reinforcement (sugared water).

Initially, during the testing of the virtual system, a linear track without turns was chosen. Running forward along this track was reinforced with sugared water (about 4 rewards per minute). In the subsequent stage of training, each animal was placed directly into the virtual T-maze (see Figure 11), where a tower (a tail triangle protruding from the maze walls) appeared at the end of either left or right arm. This setup aimed to prompt recognition and memory of the tower as a final goal for retrieving positive reinforcement in that specific location.

Additionally, we introduced black and white wall colors, which rates associated with the location of the towers at the end of the maze. White indicated the tower on the left, while black indicated the tower on the right. At the end of the training stage, the tested rats had to independently choose which of the two towers to approach, with correct choices being rewarded.

In the final stage of learning, we introduced a color wall mismatch starting from the middle of the track. The walls were white or black for the first half of the track, transitioning to grey for the assessment of working memory in rats. Depending on the wall color in the first part of the track, the rats had to recall the color and corresponding tower location to make the correct turn to the right or left, similar to the previous stage.

The entire track length remained constant at 9 m. Initially, the walls were white with black circles indicating a turn to the right and a green tower at the end. During color mismatch conditions, the first half of the track (4.5 m) had white walls with black circles, followed by grey walls for the assessment phase of working memory in rats.

**Figure 12.**
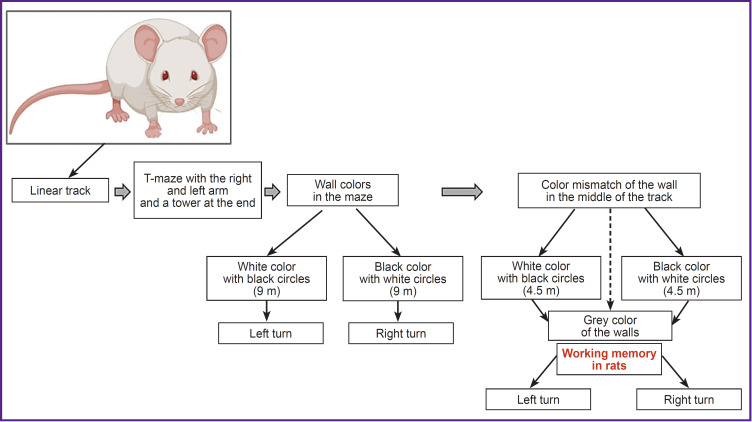
Schematic view of the developed protocol for evaluation of the working memory in the tested rats based on remembering the desired arm of the T-maze in accordance with the wall color

Transitions between stages were made when there were four or more attempts per minute, and rats achieved correct results exceeding 80% over 2–3 consecutive days. Thus, we modified the protocol for evaluating working memory in rats based on [12], developing our own experimental setup and virtual reality system. Summarizing this data, we established a sequence of actions for evaluating the rat ability to remember the correct turn to the necessary arm of the T-maze, as shown in Figure 12.

## Conclusion

In the present study, we have presented several possible versions of virtual reality systems designed to test cognitive functions of rodents. The most optimal experimental setup, which minimizes animal stress and facilitates the evaluation of working memory with a T-maze, was chosen from the presented options. Working memory was assessed by remembering the correct turn to the desired arm of the virtual T-maze based on the color of the walls and the shape of landmarks on them.

The developed experimental setup and protocol of assessing working memory in rats have significant engineering, biological, and biomedical implications. This protocol can be utilized by a wide range of research groups to investigate memory processes in rodents. Evaluating cognitive functions in experimental animals is crucial for clinical and preclinical investigations. Our system is effective for these tasks, allowing for rapid and optimal customization of specific research needs.
